# B cell maturation antigen is a novel target for immunotherapy of acute myeloid leukemia

**DOI:** 10.1186/s13045-025-01741-y

**Published:** 2025-10-24

**Authors:** Ashley Varkey, Manpreet Bariana, Mark Batistick, John Church, Elena Cassella, Shaina Anuncio, Shabnam Samimi, Alexander J. Vallone, Zephyr Hameem, Sarvarinder Gill, James McCloskey, Yiming Chen, Ming Tan, Maher Albitar, Benjamin Tycko, Kar. F. Chow, Giuditta Mantile-Selvaggi, David S. Siegel, Johannes L. Zakrzewski

**Affiliations:** 1 Center for Discovery and Innovation, Hackensack Meridian Health, 111 Ideation Way, Nutley, NJ 07110, USA; 2 Department of Pediatrics, Hackensack University Medical Center, Hackensack, NJ USA; 3 Hackensack Meridian School of Medicine, Nutley, NJ USA; 4 Stony Brook University Hospital, Stony Brook, NY USA; 5 Vitruviae LCC, Nutley, NJ USA; 6 John Theurer Cancer Center at Hackensack University Medical Center, Hackensack, NJ USA; 7 Department of Biostatistics, Bioinformatics and Biomathematics, Georgetown University, Washington, D.C USA; 8 Genomic Testing Cooperative, Lake Forest, CA USA; 9 Department of Oncology, Georgetown University, Washington, D. C USA; 10 Department of Pathology, Hackensack University Medical Center, Hackensack, NJ USA

**Keywords:** Acute myeloid leukemia (AML), T-cell engager (TCE), Immunotherapy, B cell maturation antigen (BCMA)

## Abstract

B cell maturation antigen (BCMA) has emerged as a prominent immunotherapeutic target in multiple myeloma (MM) due to its restricted expression on MM cells, plasma cells and mature B cells, with minimal presence in other normal tissues. In this study, we demonstrate through RNA sequencing and flow cytometry analyses of acute myeloid leukemia (AML) cell lines and primary patient samples that BCMA is also a relevant AML-associated antigen. Its robust surface expression on AML cells positions it as a promising candidate for targeted immunotherapy. Functionally, our findings indicate that BCMA in AML operates similarly to its role in MM – engaging the NF-kB pathway upon ligand binding, thereby activating gene expression programs that support leukemia cell survival and proliferation. We assessed several BCMA-targeted immunotherapeutic strategies, including bispecific T-cell engagers (TCE) and chimeric antigen receptor (CAR) transduced T-cells, NK-cells, and macrophages. We found that TCE treatment and BCMA CAR engineering markedly improved effector cell mediated cytotoxicity against AML cells, underscoring BCMA’s potential as a viable therapeutic target in AML. Furthermore, BCMA- directed TCE therapy significantly augmented the anti-leukemic activity of adoptively transferred CD8^+^ T-cells in a human AML xenograft model. Taken together, these findings support BCMA as a novel immunotherapeutic target in AML. Leveraging existing BCMA-directed treatments developed for MM could enable rapid clinical translation and broaden immunotherapy options for patients with AML.

**Supplementary Information:**

The online version contains supplementary material available at 10.1186/s13045-025-01741-y.

## To the editor

**Fig. 1:**
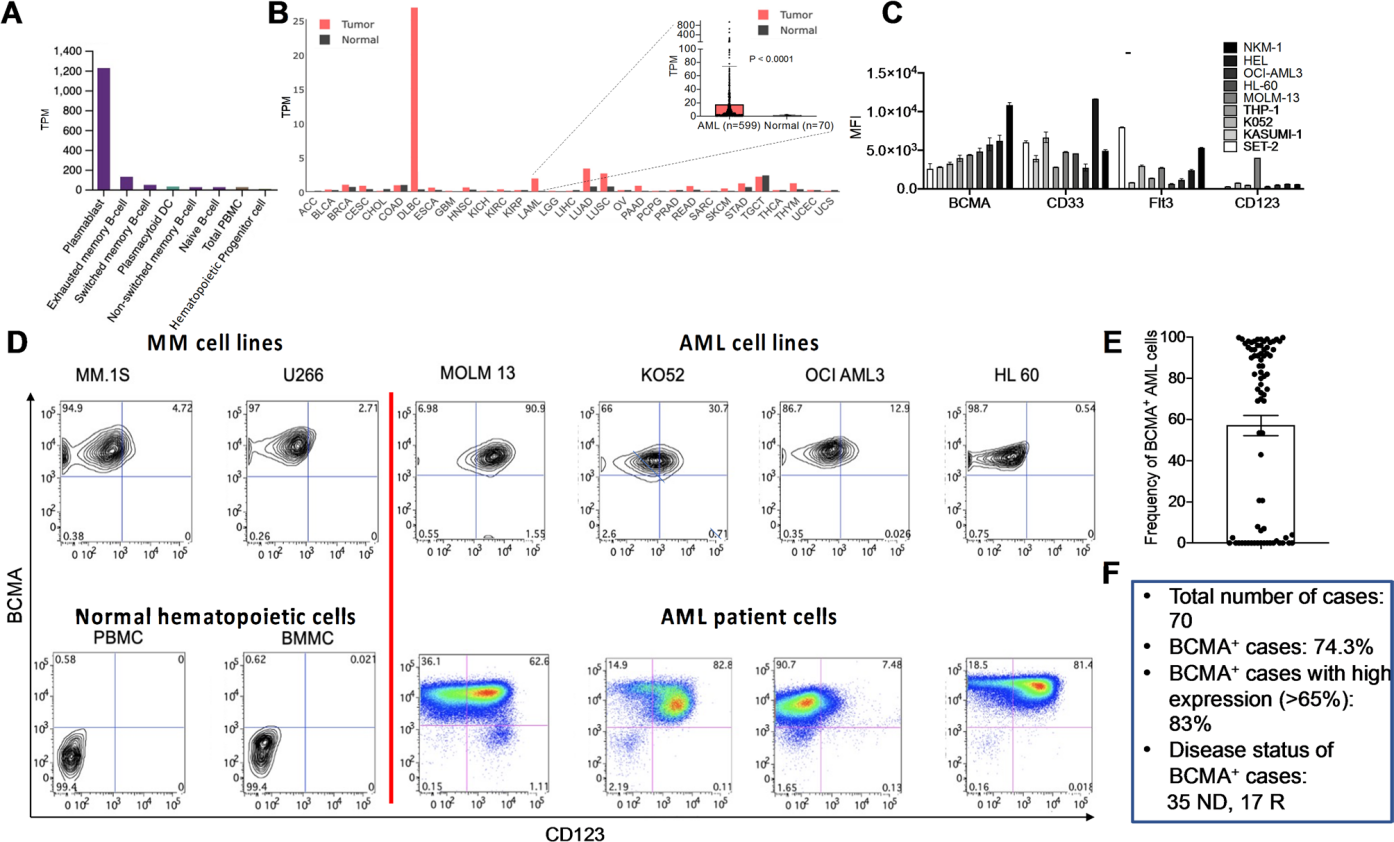
BCMA is an AML associatedtumorantigen. **(A)** Median normalized expression (transcripts per million) of BCMA in B lineage cells, PBMC and CD34^+^ HSPC based on single cell RNA-seq analysis of peripheral blood from 13 healthy individuals, created with The Human Protein Atlas using a previously published dataset (proteinatlas.org, TNFRSF17). **(B)** Median normalized expression of BCMA in 31 cancer types and paired normal tissues, created with the Gene Expression Profiling Interactive Analysis (GEPIA) platform using The Cancer Genome Atlas (TCGA) for 31 tumor types and additional in-house bulk RNA sequencing gene expression data sets for AML; 150 TCGA cases were combined with 449 in-house cases for a total of 599 AML cases, presented as a separate enlarged panel. ACC: Adrenocortical carcinoma, BLCA: Bladder Urothelial Carcinoma; BRCA: Breast invasive carcinoma; CESC: Cervical squamous cell carcinoma and endocervical adenocarcinoma; CHOL: Cholangio carcinoma; COAD: Colon adenocarcinoma; DLBCL: Diffuse Large B-cell Lymphoma; ESCA: Esophageal carcinoma; GBM: Glioblastoma multiforme; HNSC: Head and Neck squamous cell carcinoma; KICH: Chromophobe renal cell carcinoma; KIRC: Kidney renal clear cell carcinoma; KIRP: Kidney renal papillary cell carcinoma; LAML: Acute myeloid leukemia; LGG: Brain Lower Grade Glioma; LHC: Liver hepatocellular carcinoma; LUAD: Lung adenocarcinoma; LUSC: Lung squamous cell carcinoma; OV: Ovarian serous cystadenocarcinoma; PAAD: Pancreatic adenocarcinoma; PCPG: Pheochromocytoma and Paraganglioma; PRAD: Prostate adenocarcinoma; READ: Rectum adenocarcinoma; SARC: Sarcoma; SKCM: Skin Cutaneous Melanoma; STAD: Stomach adenocarcinoma; TGCT: Testicular Germ Cell Tumors; THCA: Thyroid carcinoma; THYM: Thymoma; UCEC: Uterine Corpus Endometrial Carcinoma; UCS: Uterine Carcinosarcoma. **(C)** Nine AML cell lines (THP-1, OCI-AML3, MOLM-13, K052, KASUMI-1, SET-2, NKM-1, HL-60, HEL) were analyzed for the expression of the MM-associated antigen BCMA and AML-associated antigens CD33, Flt3, and CD123. Mean and SEM are presented. **(D)** MM cell lines (MM.1 S, U266), AML cell lines (MOLM-13, K052, OCI-AML3, HL-60), healthy control cells (PBMC, BMMC) and AML cells were analyzed for expression of BCMA and CD123. Representative contour plots (cell lines and healthy control cells) and pseudocolor dot plots (AML patient cells) are presented. **(E-F)** Frequency of BCMA expression by AML cells from 70 patients. The error bar presents the Mean± SEM (**E**). High expression was defined as greater than 65% BCMA positivity among total AML cells (**F**)

Immunotherapies have transformed treatment for other blood cancers but remain ineffective in AML due to limited specific targets and AML-related immunosuppression. B-cell maturation antigen (BCMA, *TNFRSF17*), essential for B-cell and plasma-cell survival [1] is a key therapeutic target in MM due to its high expression in malignant plasma cells and limited presence in healthy tissues [2]. This study identifies BCMA as a novel AML biomarker, oncogenic pathway and immunotherapy target. Single-cell RNA-seq confirmed that BCMA expression is highest in plasmablasts, with low levels in memory B-cells and none in T-cells or CD34^+^ progenitors (Fig. 1A). TCGA RNA-seq analysis across 32 tumor types showed BCMA expression in B-cell lymphoma, AML, and select solid tumors (Fig. 1B). To further validate BCMA expression in AML, we analyzed 599 cases combined from TCGA and Genomic Testing Cooperative datasets, finding expression in 98.8% of cases across all ages and subtypes, with minimal levels in paired normal cells (Fig. 1B, Additional File 1). While our findings reveal that BCMA expression is a ubiquitous feature of AML cells irrespective of their specific genetic subtype, we identified correlations between the expression of BCMA and various genes in AML cells. The top two genes with the strongest correlation with BCMA expression in AML cells were *BOB.1* (Pearson *r* = 0.88, *p* < 0.0001) and the PD-1 ligand *PD-L2* (Pearson *r* = 0.80, *p* < 0.0001) (Additional File 2). *BOB.1* is a B cell-specific transcriptional co-activator for *Oct-1* and *Oct-2* transcription factors. Ectopic expression of *BOB.1* occurs in ~ 98% of primary AML tumor samples without predilection for a particular subtype and it is a negative prognostic factor in AML [3, 4]. Flow cytometry confirmed high BCMA surface expression levels in AML cell lines and primary cells (Fig. 1C + D, Additional Files 3–5), comparable to CD33 and higher than Flt3 and CD123. Among AML patient samples, 74.3% were BCMA-positive, with 83% of those classified as BCMA-high (greater than 65% BCMA positivity among total AML cells) (Fig. 1E + F). These findings suggest that BCMA is a prevalent and promising immunotherapeutic target in AML.

**Fig. 2:**
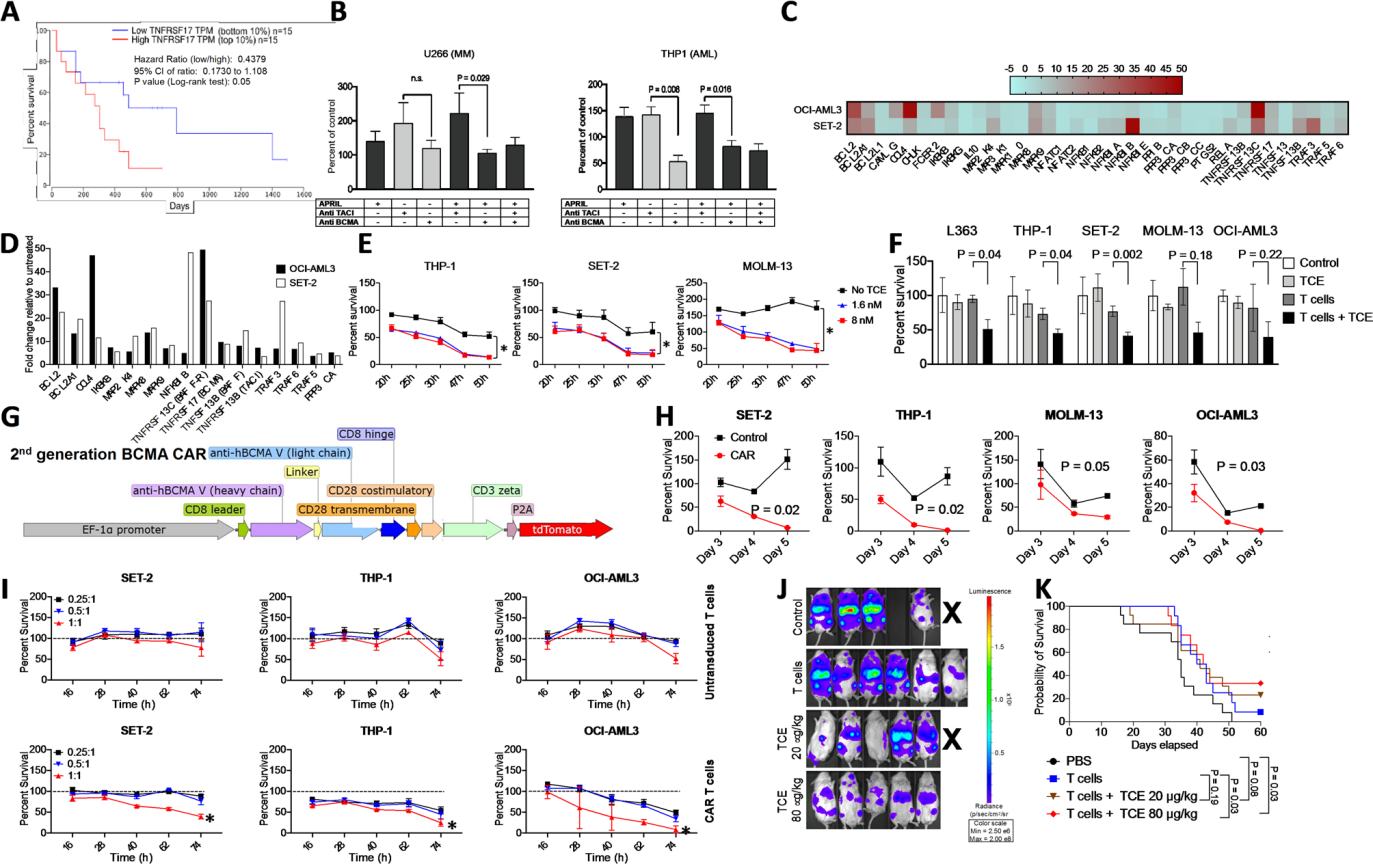
BCMApromotesAML cell survival,and it is a promising target for immunotherapy of AML. **(A)** Probability of survival comparing AML patients with the highest BCMA levels (top 10%) with patients with the lowest expression levels (bottom 10%). TCGA survival data were analyzed by Log-rank test via the oncolnc.org platform (*n* = 15 per group). **(B)** U266 and THP-1 cells were cultured in the presence or absence of APRIL (600 ng/ml), anti-TACI (10 µg/ml) and anti-BCMA (10 µg/ml) neutralizing antibodies. Cell proliferation was measured after 32 h by luciferase assays and mean and SEM of normalized values (percent of untreated control cells) are presented. Combined data from three independent experiments are presented (*n* = 9). Differences between groups were analyzed by Mann-Whitney U test. **(C + D)** BAFF/APRIL target gene expression was analyzed by real time- PCR in SET-2 and OCI-AML3 cells treated with or without BAFF + APRIL (1 µg/ml each). **(C)** A heatmap shows fold changes for all 36 genes. **(D)** The top 16 upregulated genes are shown (experiments replicated with similar results). **(E)** Luciferase transduced AML cells **(**THP-1, SET-2, and MOLM-13) were co-cultured with BCMAxCD3 TCE (1.6 nM and 8 nM) and activated CD8^+^ T cells (E: T ratio of 4:1). Killing was measured after 20, 25, 30, 47, and 50 h by luciferase assay. Normalized values (killing percentage based on untreated target cells) is shown (Mean ± SEM, (*n* = 3)). P values were calculated by 2-way ANOVA and compare target cell survival with TCE (8nM) and without TCE. The asterisk sign indicates a P value of < 0.05. **(F)** MM (L363) and AML (THP-1, SET-2, MOLM-13, OCI- AML3) luciferase-labeled cells were co-cultured with BCMA TCE (8 nM) and activated CD8^+^ T cells (E: T 4:1). Killing was measured at 30 h. Normalized killing (%) is shown (Mean±SEM, *n* = 6, two experiments combined). P values were calculated by unpaired t test and compare target cell survival with/without TCE. **(G)** Lentiviral bi-cistronic vector map for hBCMA-dtomato expression: a 2nd generation CAR with a CD28 co-stimulatory endodomain was used for CAR-T and CAR-NK cell engineering. **(H)** Luciferase transduced AML cells (SET-2, THP-1, MOLM-13, OCI-AML3) were co-cultured with CAR- macrophages or control macrophages (E: T 2:1). Killing was measured on days 3, 4, and 5. Normalized killing (%) is shown (Mean±SEM, *n* = 9, three experiments combined). P values were calculated by unpaired t test. **(I)** Luciferase- transduced AML cells (SET-2, THP-1, OCI-AML3) were co-cultured with CAR-T-cells or control T-cells at indicated **E**: T ratios. Killing was measured (16–74 h). Normalized killing (%) is shown (Mean±SEM, *n* = 9, three experiments combined). The asterisk sign indicates a P value of < 0.05 calculated by unpaired t test, comparing target cell survival in the 1:1 E: T condition (untransduced T-cells vs. CAR-T-cells). **(J)** NSG mice received 1 × 10^6^ THP-1-luc (HLA-A*0201^+^) AML cells followed 10 days later by either PBS or CD8^+^ human T cell injections (1 × 10^6^ cells/mouse) (day 0 of treatment). BCMA x CD3 TCE treatment (20–80 µg/kg) was administered twice a week for 4 weeks (*n* = 5–6 per group). AML progression was analyzed by in vivo BLI. Pseudocolor images superimposed on conventional photographs on Day 21 are presented. **(K)** Combined Survival for all 3 experiments was analyzed by the Log-rank test

BCMA’s role in AML is unclear, but survival analysis shows that patients with high BCMA expression have significantly worse outcomes than those with low expression (Fig. 2A), highlighting its potential as a therapeutic and prognostic marker. TACI and BAFF-R, alongside BCMA, are receptors for the B-cell differentiation and survival factors BAFF and APRIL. Both receptors are expressed in AML cells at the RNA/protein levels, but BCMA showed the highest tumor-to-normal expression ratio (Additional File 6). APRIL stimulation increased AML cell proliferation, which was significantly reduced by BCMA blockade, mirroring MM findings and indicating that APRIL activity is primarily mediated through BCMA (Fig. 2B). BCMA signals through NF-kB upon BAFF/APRIL binding, upregulating genes that control B-cell maturation and MM cell survival [5, 6]. We created several AML NF-kB reporter cell lines and found that BAFF/APRIL treatment induced NF-kB transcriptional activity in AML cells (Additional File 7). Targeted gene expression profiling showed a robust NF-kB–related transcriptional signature in AML cells, with upregulation of BCL2, BCL2A1, CCL4, and TRAF family genes linked to AML survival and progression (Fig. 2C + D). Collectively, these results show that BCMA mediates pro-survival/pro-proliferation signaling in AML cells via NF-kB, reinforcing its relevance as AML therapeutic target.

We evaluated several BCMA-directed immunotherapies and assessed their in vitro efficacy in luciferase-based killing assays with luciferase-transduced AML target cells. BCMA-directed TCE therapy effectively killed AML cells and MM controls (Fig. 2E + F). Interleukin-18, a cytokine enhancing CAR-T-cell function [7] further boosted TCE efficacy (Additional File 8). To broaden the therapeutic scope, we evaluated BCMA CAR-T-cells, CAR-NK-cells, and CAR-macrophages in AML killing assays, revealing that BCMA CAR expression significantly enhanced cytotoxicity in all types of effector cells (Fig. 2G–I, Additional File 9). Finally, we tested the in vivo efficacy of BCMA-directed TCE therapy in a human AML xenograft model. CD8^+^ human T-cells administered with low or high doses of BCMAxCD3 TCE significantly reduced the leukemic burden. High-dose TCE provided the most durable anti-leukemic effect and extended overall survival (Fig. 2J + K, Additional File 10). Collectively, these results support the efficacy and translational potential of BCMA-targeted immunotherapies – both T-cell dependent and independent – in treating AML.

AML immunotherapy faces challenges from disease and treatment-related T-cell impairment. However, as T-cell dysfunction in AML often improves with treatment response [8]there is a compelling rationale for evaluating T-cell-based immunotherapies in post-induction/post-consolidation settings when the disease burden is low. Additional strategies to overcome AML-associated immune challenges may include immune modulators, T cell function boosting cytokines such as IL-18^7^ and IL-7 [9], antibody-drug conjugates, and off-the-shelf allogeneic therapies like CAR-macrophages, CAR-NK-cells, and next-gen CAR-T-cells (characterized by hypoimmunogenicity and lack of alloreactivity) [10, 11]. The toxicity profile of BCMA-targeted therapies includes the depletion of some B cell subsets and plasma cells, often leading to prolonged hypogammaglobulinemia, which is associated with an increased risk of invasive infections and can be mitigated by intravenous immunoglobulin administration. Cytokine release syndrome, a common adverse event of BCMA-directed TCE and CAR T cell therapy, can be adequately managed by administration of tocilizumab or steroids. Gamma secretase inhibitors (GSI), which prevent BCMA shedding and improve BCMA CAR-T-cell efficacy in MM [12] also increase BCMA surface expression in AML (Additional File 11). However, GSI use may inhibit Notch signaling, which normally suppresses AML growth [13].

In conclusion, our study introduces BCMA as a promising immunotherapy target in AML with robust expression and a functional role in AML cell proliferation. We demonstrate potent anti-leukemic activity of BCMA-directed immunotherapies including bispecific T-cell engagers and CAR-engineered effector cells. Since immunogenicity and response to immunotherapies often correlates with tumor associated antigen density, a key next step prior to clinical translation of BCMA-directed AML immunotherapies includes the quantitative assessment of BCMA density in patient-derived AML cells. Existing BCMA targeting agents developed for MM offer a rapid path to clinical translation in AML.

## Supplementary Information

Supplementary Material 1

## Data Availability

For original data, please contact johannes.zakrzewski@hmh-cdi.org. RNA-seq data are available at [https://www.cancer.gov/tcga](https:/www.cancer.gov/ccg/research/genome-sequencing/tcga) and from the Genomic Testing Cooperative.

## Abbreviations

BCMA: B cell maturation antigen
MM: Multiple myeloma
AML: Acute myeloid leukemia
CAR: Chimeric antigen receptor
TCE: T cell engager
ALL: Acute lymphoblastic leukemia
FDA: Food and Drug Administration
APRIL: A proliferation inducing ligand
BAFF: B cell activating factor
PBMC: Peripheral blood mononuclear cells
PBS: Phosphate- buffered saline
FBS: Fetal bovine serum
IL-2: Interleukin-2
NK: Natural killer
BMMC: Bone marrow mononuclear cells
TCGA: The Cancer Genome Atlas
GEPIA: Gene Expression Profiling Interactive Analysis
UALCAN: University of Alabama at Birmingham Cancer data analysis Portal
TPM: Transcripts per million
IACUC: Institutional Animal Care and Use Committee
NSG: NOD/ SCID/ IL2Rg(null)
BLI: Bioluminescence imaging
TNFRSF17: Tumor necrosis factor receptor superfamily
GTC: Genomic Testing Cooperative
FAB: French- American- British classification system
PCR: Polymerase chain reaction
TRAF: Tumor necrosis factor receptor associated factors
