# Supplementary material for: B cell maturation antigen is a novel target for immunotherapy of acute myeloid leukemia
Source: J Hematol Oncol. 2025 Oct 24;18:89. doi: 10.1186/s13045-025-01741-y (PMC12553267; doi:10.1186/s13045-025-01741-y)
Supplement: Supplementary file 1 — Supplementary Material 1 [file 13045_2025_1741_MOESM1_ESM.docx]

**Additional Methods and Figures**

**Materials and Methods**

**Cell lines**

Vendor-authenticated MM cell lines (U266, MM.1S) and AML cell lines (THP-1, OCI-AML3, MOLM-13, K052, KASUMI-1, SET-2, NKM-1, HL-60 and HEL) were originally obtained from the ATCC (Manassas, VA) in 2018. These cells were cultured in either RPMI1640 or MEM-α medium supplemented with 10% heat-inactivated FBS (GIBCO, Gaithersburg, MD), 1% L-glutamine, and penicillin–streptomycin (Corning, Corning, NY) in a humidified environment of 5% CO_2_ at 37^o^C. Luciferase-expressing variants were obtained by transduction with a third-generation lentiviral vector with bi-cistronic expression of dTomato and luciferase (pUltra-Chili-Luc; Addgene #48688) (Addgene, Watertown MA). U266-GFP-Luciferase cells were kindly donated by Dr. Eric Smith (Memorial Sloan Kettering, NY). All cell lines were used for experiments within 3 weeks of thawing (up to 4 passages) and were annually tested for Mycoplasma negativity using a Mycoplasma PCR Kit from Lonza (Watersville, MD).

**Primary cells**

Peripheral blood mononuclear cells (PBMC) were isolated from the whole blood of healthy volunteers. Phosphate-buffered saline (PBS) + 2% fetal bovine serum (FBS) was used to dilute the blood at a 1:1 ratio, and then, the mixture was slowly added to 15 ml of Lymphoprep density gradient medium in a SepMate tube (STEMCELL Technologies, Vancouver, Canada). The tubes were centrifuged at 1200*g* for 10 min at room temperature to segregate the PBMCs, which would be enriched in the top layer. The PBMC were collected into new tubes and washed two times with cold PBS + 2% FBS. T cells were expanded from PBMC after seven days of incubation with human anti-CD3/anti-CD28 T cell activation beads (Proteintech Group Inc, Rosemont, IL) and 50 IU/ml of human interleukin -2 (IL-2) (Peprotech, Cranbury NJ) at a cell to bead ratio of 1:1. This protocol routinely yielded CD3^+^ T cells of greater than 95% purity as determined by flow cytometry (data not shown). The beads were removed via magnetic separation and cells were suspended in freezing medium (CryoStor® CS10, STEMCELL Technologies) and stored at −80°C till further use. The activated PBMC, better classified as PBMC-derived activated T cells, were thawed a week before the assay in RPMI1640 supplemented with 10% heat-inactivated FBS with IL-2 and co-incubated with activation/expansion beads at a ratio of 1:1. Natural killer (NK) cells were expanded from PBMC over a period of seven to 10 days using the ImmunoCult NK Cell Expansion Kit from STEMCELL Technologies). This protocol routinely yielded CD56^+^ T cells of greater than 95% purity as determined by flow cytometry (data not shown). Primary bone marrow mononuclear cells (BMMCs) were purchased from Miltenyi (Gaithersburg, MD). AML patient cells were obtained for diagnostic flow cytometry at the John Theurer Cancer Center of HUMC and anti-BCMA was included in the standard-of-care leukemia panel. Eight AML patient cell samples were purchased from the Fred Hutchinson Cancer Center/University of Washington Hematopoietic Disease Repository. Patient samples were collected after informed consent was obtained and their use for research was approved by the Institutional Review Board (IRB) (protocol# RG1000570 at Fred Hutchinson Cancer Center and protocol# Pro2018-1022 at Hackensack University Medical Center).

**Transcriptome profiling of BCMA expressing cancer cells**

BCMA expression data by cancer cells were in part based upon data generated by The Cancer Genome Atlas (TCGA) Research network ([https://www.cancer.gov/tcga](https://www.cancer.gov/ccg/research/genome-sequencing/tcga)) combined with Gene Expression Profiling Interactive Analysis (GEPIA) (gepia.cancer-pku.cn)^17^ and The University of Alabama at Birmingham Cancer data analysis Portal (UALCAN) (ualcan.path.uab.edu)^18^. BCMA expression in normal hematopoietic cells was analyzed by single cell RNA sequencing utilizing the Human Protein Atlas v24.0 (proteinatlas.org) and a dataset published by Monaco et al^19^. Median normalized expression of BCMA in AML cells (transcripts per million (TPM)) were analyzed via each respective database. Further RNA sequencing data from 449 AML patients, 290 MM and 201 diffuse large B cell lymphoma (DLBCL) patients at Hackensack University Medical Center was obtained from the Genomic Testing Cooperative using a blood cancer targeted RNA sequencing panel^20, 21^.

**Probability of survival with BCMA expression in AML**

TCGA survival data via the oncoInc.org platform^22^ was utilized to analyze survival probabilities associated with BCMA expression in AML by Log-Rank test.

***In vitro* cytotoxicity assays**

For *in vitro* assays, several subsets of effector cells including PBMC derived CD8^+^ activated T cells, CAR T cells, CAR NK cells, and CAR macrophages, were cocultured with luciferase-expressing AML target cells at indicated effector-to-target (E:T) ratios. At the indicated timepoints over 72 h, luminescence intensity measurements were obtained by adding luciferin (1 μL of a 15mg/mL solution per 100 μL of media) (Goldbio, St Louis, MO) and measuring luminescence intensity with a Tecan M Plex Pro 200 plate reader (Tecan, Switzerland). All the quantitative results were obtained from at least three independent runs with three technical replicates each.

In T cell engager (TCE) assays, 5 × 10^3^ luciferase-transduced AML cells (THP-1, SET-2, MOLM-13 and OCI-AML3) were cultured with effector cells (PBMC derived activated CD8^+^ T cells) at the specified E:T ratios for up to 72 hours. BCMA-directed TCE purchased from BPS Bioscience (San Diego, CA) was added to the media at the desired concentrations. Target cell monocultures were used as controls to establish baseline survival of each target cell type. Target cell survival was determined by adding luciferin reagent at the specified time points followed by measurement of luminescence via a multimodal plate reader. These assays were also replicated with the addition of IL-18 (50 ng/ml) (R&D systems, Minneapolis, MN) to the co-culture of 2 AML cell lines (SET-2, OCI-AML3) with PBMCs derived activated T cells (E:T- 6:1) and the BCMA X CD3 TCE.

CAR based cytotoxicity assays were performed with 5 x 10^4^ luciferase transduced tumor target cells (THP-1, SET-2, MOLM-13, and OCI-AML3) seeded per well. The effector cells (CAR T cells, CAR NK cells or CAR macrophages) were added at E:T ratios ranging from 0.25:1 to 2:1. Target cell survival at desired time points were assessed by measurement of luminescence via multimodal plate reader and the percent survival was calculated by normalizing data to target cell monocultures.

**APRIL BCMA Proliferation Assays**

Proliferation of AML cells (THP-1, SET-2) was measured in the presence of BCMA ligand APRIL with multiple myeloma cells (U266) as negative control. 1 × 10^4^ cells were cultured in the presence or absence of human recombinant protein APRIL (Peprotech; 600 or 120 ng/ml) along with neutralizing antibodies anti-TACI (R&D systems; 10 mg/ml) or anti-BCMA (Creative Biolabs, Shirley, NY; 10 mg/ml). Cell proliferation was evaluated by luciferase assays by luminescence measurements via a multimodal plate reader.

**BCMA NF-kB reporter assay**

NF-kB transcriptional reporter AML cell lines were obtained by transduction with pGreenFire 2.0 NFκB Reporter Lentivector from System Biosciences. 5-8 × 10^4^ NF-kB transduced tumor cells (U266, THP-1, SET-2, and OCI-AML3) were seeded per well and co-cultured with human recombinant BAFF (1,2 or 4 mg/ml) and APRIL (1 or 2 mg/ml) proteins (Peprotech). NF-kB transcriptional activity was assessed by measurement of luminescence via multimodal plate reader and the percent cell survival was calculated by normalizing data to NF-kB transduced tumor cells without BAFF and APRIL.

**Mouse Models**

All animal studies were conducted in accordance with Institutional Animal Care and Use Committee (IACUC) standards and with IACUC approval (protocol#24-20). Eight to ten-week-old immunodeficient NOD/SCID/IL2Rg(null) (NSG) mice purchased from the Jackson Laboratory (Bar Harbor, ME, USA) were used for AML xenograft experiments.

For the AML xenograft model, 1x10^6^ THP-1-tdtom-Luc cells were injected intravenously in eight NSG mice after sublethal irradiation (200 cGy) and bone marrow engraftment of AML cells was confirmed by *in vivo* bioluminescence imaging (BLI) prior to assignment to control and treatment groups. On day 10 after tumor cell injections, 1.5 × 10^6^ human CD8^+^ T cells or PBS were injected retro-orbitally (treatment day 0). On day 12, mice were treated with PBS or BCMA x CD3 TCE at 20 ug/kg or 80 ug/kg intravenously twice a week for four weeks. Animals were monitored daily for survival and euthanized when they reached clinical endpoints (hindlimb paralysis).

**Antibodies and flow cytometry**

For cell analysis of surface markers (BCMA, CD123, Flt3, CD33, CD38), MM and AML cell lines were stained with human anti-BCMA-BV421 (BioLegend (San Diego, CA), 19F2)), anti-CD123-PE (Beckman Coulter (Jersey City, NJ), SSDCLY107D2)), anti-FLT3-PE-Cy7 (BioLegend, BV10A4H2), anti-CD268-PE BAFF-R (BioLegend, 11c1), anti-CD267- APC TACI (BioLegend, 1A1), and anti-CD33 (Absolute Antibody (Oxford, UK), hP67.6 (Gemtuzumab)) with Mouse Anti-hIgGFc-FITC (Southernbiotech (Birmingham, AL), JDC-10)) as a secondary antibody for 20 minutes at 4°C in PBS with 0.5% (w/v) bovine serum albumin (PBS/BSA), fixed in 2% (w/v) paraformaldehyde (PFA) in PBS for 15 minutes, washed, and resuspended in PBS/BSA prior to acquisition. Gamma secretase inhibitor treatment was performed with the compound RO4929097 (Selleck Chemicals, Houston TX) at 1 μM for 24 hours. All flow cytometry was performed on a LSRFortessa™ Cell Analyzer (BD) and data were analyzed with Flowjo software version 10.5.3 (BD).

**Plasmid construction and virus production**

The first-generation CAR sequence was composed of a CD8 leader sequence, a humanized BCMA single-chain variable fragment (scFv), as per patent number US 20210277136A1, a CD8 hinge, a CD28 transmembrane domain and a CD3zeta activating domain. The second-generation CAR sequence also contained a CD28 co-stimulatory domain between transmembrane and activating domains. The sequence was inserted into a bicistronic lentiviral vector under control of EF-1alpha promoter, also expressing dtomato for visualization and selection of transduced cells. Viral particles were produced by transient transfection of Lenti-X 293T cells with the CAR-expressing plasmid, psPAX2 packaging plasmid and either pLTR-RD114A plasmid encoding RD114 envelope (for transduction of NK cells) or pVSV-G (for transfection of THP-1 cells). On day 2 post transfection, the viral supernatant was harvested, filtered with 0.45 um filter and used for transduction. For T cells only, the second-generation CAR sequence was inserted in SFG vector and fused with a terminal GFP tag. Viral particles were produced by transient transfection of GP2-293 cells with the CAR-expressing plasmid and pVSV-G. On day 2 post transfection, the viral supernatant was harvested, filtered with 0.45 um filter and used for transduction.

**Viral transduction of T cells**

Human T cells were activated and expanded from PBMCs with human anti-CD3/anti-CD28 T cell activation beads (Proteintech) (bead/cell ratio of 1:1) and 50 IU/mL IL-2 (Peprotech) and cultured in RPMI1640 supplemented with 10% FBS, 2 mM L-glutamine, and 1% penicillin/streptomycin. After 1 week of expansion, T cells were transduced by spinoculation (2000x G, 33^o^ C, 60 min) on plates coated with retronectin (10 mg/cm^2^) (Takara Clontech) with retroviral supernatant from viral packaging cells. T cells were rested for 2 days and then used for in-vitro assays.

**Viral transduction of THP-1 cells and THP-1 differentiation into macrophages**

THP-1 cells were also transduced by spinoculation (2000x G, 33^o^C, 60 min) in 24-well retronectin coated plate. The cells were allowed to grow for 7 days then sorted to obtain dtomato+ cells that stably integrated the lentiviral vector. Untransduced and CAR transduced THP-1 cells were differentiated into macrophages by 48-hour incubation in the presence of 100 ng/ml of phorbol 12-myristate 13-acetate (PMA) in a complete medium. After 48h incubation, PMA was removed, and adherent cells were harvested by a combination of trypsin treatment and gentle scraping and used for experiments.

**Viral transduction of NK cells**

Virus used for NK cells was concentrated using Lenti-X concentrator (Takara) and resuspended in 1 ml of NK cells complete medium. 1 million peripheral blood derived primary NK cells were transduced by spinoculation (2000x G, 33 degree C, 60 min) in 24-well retronectin coated plate at a density of 500,000 cells/well and 500 ul of virus per well. NK cells were rested for 2 days in the same plate and then used for in-vitro assays.

**qPCR of BAFF/ APRIL target genes**

SET-2 and OCI-AML3 cell lines were cultured in their respective medium (RPMI + 10% FBS for SET-2 and MEM alpha + 10% FBS for OCI-AML3) with and without BAFF 1 mg/mL and APRIL 1 mg/mL. Cells were frozen after 4 hours. Total RNA was extracted from frozen cell pellets using Aurum Total RNA kit from Bio-Rad, and 2 ul of the extracted RNA was used for cDNA synthesis (iScript kit from Bio-Rad Laboratories, Hercules, CA). cDNA was used to perform real-time quantitative PCR using a predesigned BAFF/APRIL signaling panel for use with SYBR Green (Bio-Rad). Results were analyzed with PrimePCR software (GeneStudy_1.0.030.1023) to determine the upregulated genes in both cell lines.

**Assessment of Tumor Burden**

The tumor burden of individual mice was determined by measuring the bioluminescent signal intensity of tumor-bearing mice using an IVIS Lumina X5 optical imaging platform and Living Image 4.7.3 software (Revvity Inc., Waltham, MA, USA). We superimposed pseudocolor images showing the whole-body distribution of the bioluminescent signal intensity on grayscale photographs and determined total flux (photons/sec/cm^2^/sr) for individual mice.

**Statistical analysis**

All statistical analyses were done using Graph-Pad Prism version 8 for Mac (GraphPad Software, San Diego). Error bars in the presented data are the standard error of the arithmetic mean (SEM). Survival data were analyzed with the Kaplan−Meier method and log-rank test. Analysis of longitudinal tumor cell survival *in vitro* or tumor growth *in vivo* was performed by nonparametric analysis of variance (ANOVA) and Mann−Whitney test for pairwise comparisons. In the event of incomplete follow-up data, we used a more powerful repeated measures model designed for longitudinal tumor studies. For non-survival pointwise analyses comparing two groups, unpaired t test was used for comparisons assuming the same standard deviation and the nonparametric Mann−Whitney test was used for non-Gaussian distributions. A p value of less than 0.05 was considered statistically significant.

**Additional Files**

**Additional File 1**

******

*BCMA is expressed across AML subtypes*. Median normalized expression of BCMA in AML cells based on the French-American-British (FAB) classification. The figure was created with the University of Alabama at Birmingham Cancer data analysis Portal (UALCAN) platform using TCGA bulk RNA sequencing gene expression data sets.

**Additional File 2**

*TNFRSF17 (BCMA) gene expression correlates with POU2AF1 (BOB.1) and PDCD1LG2 (PD-L2) gene expression in AML patient cells.* Positive correlation in mRNA expression levels between TNFRSF17 and POU2AF1 proteins (A) and between TNFRSF17 and PDCD1LG2 proteins (B) in a cohort of 449 AML patient cell samples was measured by Pearson correlation coefficient.

**Additional File 3**

*BCMA is an AML associated antigen with expression levels similar to CD33.* PBMC (n=2), BMMC (n=2) and AML patient cells (n=8) were analyzed for CD123, Flt3, BCMA, and CD33 expression. Mean and SEM of median fluorescence intensities (MFI) is presented.

**Additional File 4**

*AML patient samples display variable BCMA and CD123 expression.* Eight AML patient samples with varying cytogenetics and mutations were analyzed for CD123 and BCMA expression and pseudocolor dot plots are presented.

**Additional File 5**

*AML patient samples display variable BCMA and Flt3 expression.* Eight AML patient samples with varying cytogenetics and mutations were analyzed for Flt3 and BCMA expression and pseudocolor dot plots are presented.

**Additional File 6**

*AML and normal cells demonstrate differential expression of BCMA, BAFF, and TACI.* (A) Comparison of BCMA, BAFF and TACI receptor expression levels between tumor (T) and normal (N) cells via Gene Expression Profiling Interactive Analysis (GEPIA) platform. The density of color in each block represents the median expression value of each gene, normalized by the maximum median expression value across all blocks. Data are displayed using a log2 scale (n=150). (B) TACI and BAFF-R expression levels in MM (MM.1S, U266, NCI-H929) and AML (K562, THP-1, MOLM-13, OCI-AML3, KO52, SET-2, KASUMI-1, NKM-1, HL-60, HEL) cells were measured by flow cytometry and are presented as mean± SEM of the percentage of positive cells.

**Additional File 7**

*Effects of BAFF and APRIL on NF-κB transcriptional activity in AML cell lines*. THP-1, SET-2 and OCI-AML3 NF-κB transcriptional reporter cells cultured in complete media with and without BAFF (1 μg/ml) and APRIL (1 μg/ml) supplementation. NF-κB transcriptional activity was analyzed by luminescence measurement. Mean± SEM of combined data from two independent experiments are presented (n=6).

**Additional File 8**

*IL-18 enhances BCMA targeted T cell killing of AML cells.* THP-1-luc and OCI-AML3-luc were co-cultured with BCMA x CD3 TCE (3.2 nM), PBMC-derived activated CD8^+^ T cells at an E:T ratio of 6:1 and comparing culture conditions +/– hIL-18 (50 ng/ul). Target cell numbers were measured at 40 h by luciferase assay. Normalized values (killing percentage based on untreated target cells) are presented (Mean±SEM). P values refer to comparisons of target cell RLU with and without the hIL-18. One of three independent experiments is presented.

**Additional File 9**

*Evaluation of BCMA CAR-NK-Cell cytotoxicity against AML cells.* SET-2, THP-1 and OCI-AML3 were cocultured with BCMA CAR-engineered PBMC-derived NK cells or untransduced control NK cells at an effector:target ratio of 1:1. Target cell numbers were measured at 28 h, 40 hand 52 h by luciferase assay. Normalized values (killing percentage based on untreated target cells) are presented (Mean±SEM). One of two independent experiments is shown (n=3).

**Additional File 10**


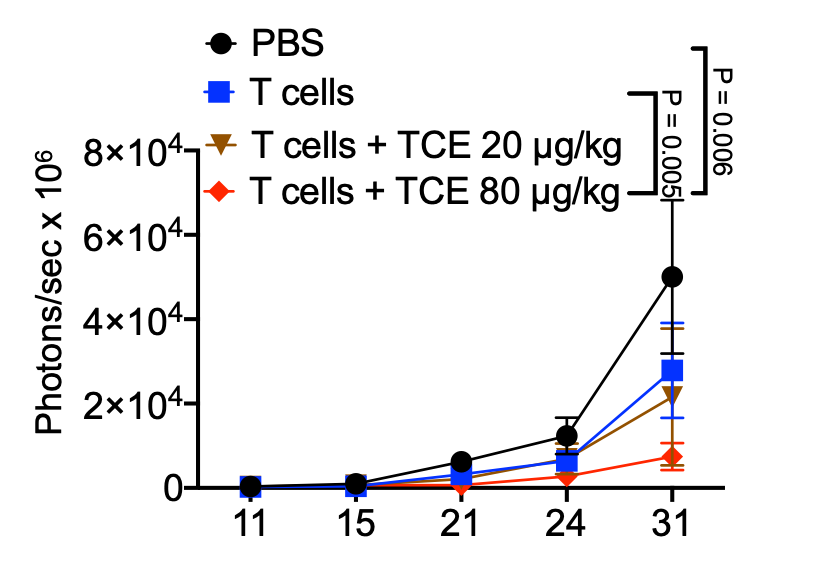


*BCMA targeted TCE therapy decrease tumor burden in a xenograft model of AML.* Mean and SEM of bioluminescence intensities of one of three independent experiments is presented. Analysis of longitudinal tumor growth was performed by nonparametric analysis of variance (ANOVA) and Mann−Whitney test for pairwise comparisons.

**Additional File 11**

*GSI treatment increases BCMA levels on the surface of AML and MM cell lines.* Four MM cell lines (MM.1S, U266, NCI-H929, L363) and eight AML cell lines (THP-1, SET-2, MOLM-13, NKM1, HEL, HL-60, KASUMI-1, KO52) were cultured for 24 hours in the presence and absence of 1 μM of the gamma secretase inhibitor (GSI) RO4929097. Mean BCMA expression levels (median fluorescence intensities (MFI)) are presented.
